# Development of a novel automatable fabrication method based on electrospinning co electrospraying for rotator cuff augmentation patches

**DOI:** 10.1371/journal.pone.0224661

**Published:** 2019-11-14

**Authors:** Sergi Rey-Vinolas, Oscar Castaño, Leonardo Ruiz-Macarrilla, Xavier Llorens, José M. Mora, Elisabeth Engel, Miguel A. Mateos-Timoneda

**Affiliations:** 1 Biomaterials for Regenerative Therapies, Institute for Bioengineering of Catalonia (IBEC), The Barcelona Institute of Science and Technology (BIST), Barcelona, Spain; 2 CIBER en Bioingeniería, Biomateriales y Nanomedicina (CIBER-BBN), Madrid, Spain; 3 Serra Hunter Fellow, Electronics and Biomedical Engineering Department, University of Barcelona (UB), Barcelona, Spain; 4 Bioelectronics Unit and Nanobioengineering Lab., Institute for Nanoscience and Nanotechnology of the University of Barcelona (IN2UB), Barcelona, Spain; 5 Servei de C.O.T., Hospital Universitario Germans Trias i Pujol, Badalona, Spain; 6 Fundació Joan Costa Roma, Consorci Sanitari de Terrassa, Terrassa, Spain; 7 Servei de C.O.T., Hospital de Terrassa, Consorci Sanitari de Terrassa, Terrassa, Spain; 8 Department of Materials Science and Metallurgical Engineering, EEBE campus, Technical University of Catalonia (UPC), Barcelona, Spain; Massachusetts Institute of Technology, UNITED STATES

## Abstract

Rotator cuff tear is one of the most common shoulder diseases. Rotator cuff augmentation (RCA) is trying to solve the high retear failure percentage after the surgery procedures (20–90%). The ideal augmentation patch must provide a temporal mechanical support during the healing process. In this work, we proposed a simple method for the fabrication of synthetic RCA patches. This method combines the use of electrospraying to produce poly-L-lactic-co-ε-caprolactone (PLC) films in an organogel form and electrospinning to produce poly(lactic) acid (PLA) nanofibers. The device consists in a combination of layers, creating a multilayered construct, enabling the possibility of tuning its mechanical properties and thickness. Besides, both techniques are simple to escalate for industrial production. A complete characterization has been performed to optimize the involved number of layers and production time of PLC films and PLA nanofibers fabrication, obtaining a final optimal configuration for RCA devices. Structural, mechanical and suture properties were evaluated. Also, the possibility of surface functionalization to improve the bioactivity of the scaffold was studied, adding aligned electrospun PLA nanofibers on the surface of the device to mimic the natural tendon topography. Surface modification was characterized by culturing adult normal human dermal fibroblasts. Lack of toxicity was detected for material presented, and cell alignment shape orientation guided by aligned fibers, mimicking tendon structure, was obtained. Cell proliferation and protein production were also evaluated.

## Introduction

Rotator cuff augmentation (RCA) has become an increasing technique to prevent tendon retear after a surgery repair. Only in the United States 17 million people suffer diseases related to rotator cuff [1–3], and 75.000 repair procedures per year are performed [4,5]. Specially, in cases with large, massive and chronic tears, the retear failure incidence ranges from 20% to 90% depending on the case [6–9].

Different types of patches for RCA have been studied since the 90s [10] in order to improve tendon reattachment and healing process, especially in large tears and chronic failures. Among them, decellularized extracellular matrix (ECM) membranes from different donors (porcine, equine, and human) provide a temporal and suitable support for cells and a source of biochemical signals and proteins that can stimulate the tissue regeneration.

However, one of the principal issues of these biological membranes is the remaining DNA. Some studies have shown the negative effects that they can produce if the decellularization and washing steps are not fully complete [11], and their possible relation with immune/inflammatory responses [12,13]. Other concern about the use of ECM membranes are their fast body resorption, losing their mechanical supporting resistance at early healing stages [14].

As an alternative to ECM membranes without antigenicity and inflammatory issues, synthetic degradable patches based on biocompatible polymers have been postulated. Properties like degradation rate, mechanical properties, shape and geometry can be easily modulated using different polymers, concentrations and fabrication techniques. In addition, many studies have reported the possibility of loading the synthetic degradable patches with signaling growth factors and drugs for their controlled release promoting a faster healing [15,16]. Depending on the fabrication method, it is possible to mimic the porosity and morphology of biological ECM. Electrospinning and electrospraying are techniques based on the same principle, the acceleration of a polymer solution through a syringe to a collector under a high electrical field to obtain micro/nano fibers and particles/films, respectively [17,18]. Electrospinning/electrospraying are widely used techniques in tissue engineering and regenerative medicine to obtain ECM-like shaped scaffolds, for example in bone regeneration [19], or as a drug delivery system [20].

In this work, we present a methodology based on the combination of electrospinning and electrospraying, to obtain synthetic and degradable patches for RCA. Electrospraying allows the fabrication of organogel films of Poly-L-lactic-co-ε-caprolactone (PLC). Previously, our group reported the fabrication methodology and characterization of scaffolds made of Poly(lactic) acid/EtLac organogels [21]. According to the expertise of clinicians the RCA scaffold device should stay for eight weeks in the damaged tendon. The goal is to provide a temporal mechanical support sharing the loads demands and allow the diffusion of oxygen and nutrients through them to enhance the healing of the injured tendon, without the need of cell infiltration at the earlier stages. After degradation and during the process of remodeling, cells are expected to penetrate within the scaffold. PLC organogel films were selected for this application due to their nanofibrous morphology and degradation properties.

Random PLA nanofibers fabricated by electrospinning were alternated between the different electrosprayed films to modulate the final desired mechanical properties of the RCA patches. This combination of semi-automated techniques will allow a large-scale patch production. The choice of PLA nanofibers as reinforcement not only affects the mechanical properties, but also its degradation products (i.e. lactate) influencing cell behavior [22,23].

Due to the high boiling temperature of EtLac (154ºC) in comparison with other common further used for electrospraying process, like Chloroform (61°C), or Trifluoroethanol (78°C), and the high pump rate, EtLac is not completely evaporated during the process making possible to obtain a polymer film in the organogel form. On the other hand, the release of lactate has been proposed to improve angiogenesis, a crucial process for tendon regeneration [24]. Hence, we have tested the biological effect of aligned PLA nanofibers on the surface of the patch.

## Materials and methods

### Materials

Poly-L/DL lactic acid 95/5 (Purasorb PLDL, intrinsic viscosity 6.15 dl/g) (PLA), and Poly-L-lactic-co-ε-caprolactone 70/30 (Purasorb PLC 7015, inherent viscosity midpoint 1.5 dl/g) (PLC) were purchased from Purac Biomaterials (The Netherlands). Ethyl L-lactate (EtLac), and 2,2,2-trifluroethanol (TFE)were purchased from Sigma-Aldrich and Panreac, respectively.

### Production of PLC films and PLA nanofibers

Polymeric films were obtained by electrospraying using a conventional equipment. Briefly, PLC solution (15% w/v in EtLac) was prepared and loaded into a 10ml syringe. The pumping syringe rate was fixed at 10 mL/h through a metallic needle (Red 25G, Nordson EFD), connected to voltage (8 KV) for accelerating the polymeric solution to the grounded collector at 10–18 cm distance. The speed of the cylindric collector was fixed at 90 rpm.

For the fabrication of PLA nanofibers, a PLA solution (4% w/w in TFE) was prepared and loaded into a 10 mL syringe. The pumping rates was fixed at 0.5 mL/h using the same needle of 25G. Distance between tip to collector was fixed at 10 cm, and collector speed was set at 90 rpm to obtain a random orientation of the nanofibers. To mimic tendon structure, aligned PLA nanofibers were also produced with the same parameters as random PLA fibers but increasing the rotator speed up to 1000 rpm. This layer was the base of the final construct. The followed protocol to produce fibers was similar to the described previously by the group [22,25].

**Fig 1:**
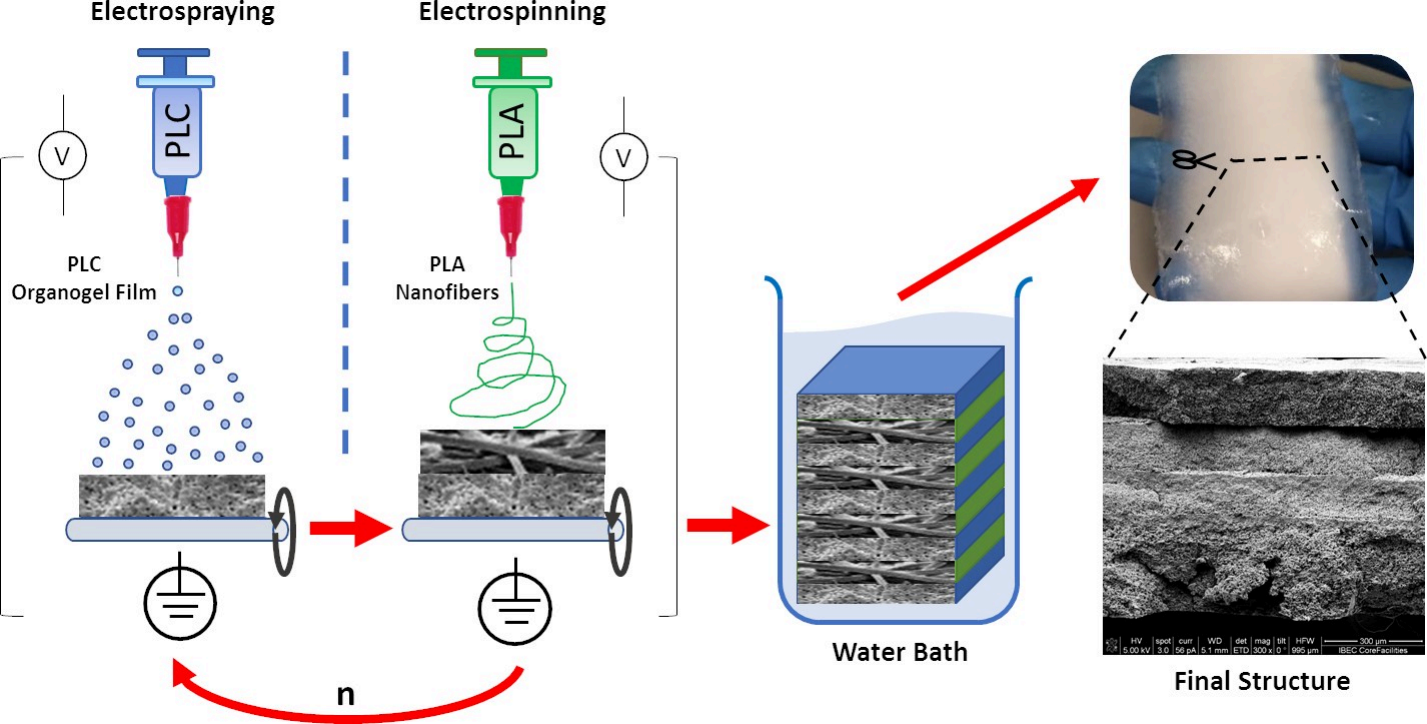
Schematic workflow representation of the RCA device fabrication.

Finally, the construct was immersed in a water bath facilitating water-solvent exchange and promoting polymers precipitation to obtain the final structure (Fig 1).

### Mechanical properties

Tensile tests were performed in a Zwick/Roell BT1 FR0.5TN.D14 testing machine. Software used for data acquisition was the provided by the manufacturer, testXpert II V3.41. The assay speed and preload of the sample were set at 10 mm/min and 0.1 N. All samples were cut into rectangles of 3x1 cm, clamping 1 cm of each sample side for testing.

Suture test was carried out with the same parameters as for the tensile tests. The sample dimensions were 3x1 cm, where the bottom side was clamped directly, and the upper side was attached the clamp through a suture retention loop (Polyglycolic acid USP 2 metric 5, Aragó). For wet condition, a 4L bath from Zwic/Roell was used filled with phosphate buffered solution (PBS) maintained at 37°C with a circulant water heater (E100, Lauda). Approached Young Modulus, and deformation at Yield Strength were evaluated. Due to the water batch set-up, deformation was assessed by the diagram displacement of testing machine.

### Structure characterization by FE-SEM

The inner structure of the fabricated RCA devices was characterized by Field Emission Scanning Electron Microscopy (FE-SEM) using a Nova NanoSEM 230 microscope (FEI Corporation). Prior to visualization, samples were freeze dried (Alpha 1–4, Christ), and sputtered with 10 nm gold layer. Images were taken at 5 KV voltage with ETD detector. Fiber diameter was measured by ImageJ software from FE-SEM images obtained.

### Biological characterization

Adult Normal Human Dermal fibroblasts (NHDF) (PromoCell, Germany) were used for the biological characterization. NHDF were cultured in Dulbecco’s Modified Eagle Medium (DMEM), supplemented with 1% L-Glutamine, 1% Penicillin/Streptomycin, and 10% Fetal Bovine Serum (FBS), all purchased from Thermo Fischer Scientific (Massachusetts, USA). Cell culture media was changed every two/three days and the cells were used in passages 3–6 for all the experiments.

Two different conditions were studied: in the first condition, RCA devices with aligned fibers on the surface were tested; whereas in the second condition, the RCA devices do not present aligned fibers on their surface. Scaffolds were cut into squares of 1x1 cm for the biological characterization assays. RCA devices without cells were used as negative controls for the experiments.

### Cytotoxicity assays

Cytotoxicity was evaluated indirectly using conditioned media assay. In parallel, 10 x 10^3^ NHDFs were seeded into 24 well plate, and scaffolds with/without PLA aligned fibers (1x1cm) were immersed in cell culture media into different 24 well plate and incubated for 24 hours. Then, the media from NHDFs was changed for conditioned media and incubated for 24 hours more. After this time, live/dead staining, based on Calcein-AM and propidium iodide (both from Sigma Aldrich), was performed. Images were taken at 10x augments with DM IRBE Leica microscope. For quantitative LDH cytotoxic evaluation, same procedure with conditioned media was used prolonged in time. At 1, 3, 5, 7 and 9 days, conditioned media in contact with materials was transferred to NHDFs wells, and material wells were replaced with new media. One condition of NHDFs incubated with media that was not in contact with materials was used as a control. LDH absorbance was read following the manufacturer’s protocol (Roche), at 492nm with a reference at 680nm with a microplate reader (Benchmark Plus, BioRad). Results are expressed as an absorbance percentage of conditioned media from materials condition over control condition.

### Cell metabolic assay

Alamar blue (Thermo Fischer Scientific) assay was selected for cell metabolic assay as non-destructive assay. Both conditions, with and without aligned fibers on top, were evaluated. Scaffolds of 1x1 cm in size were cut and placed into 24 well plates with ultra-low attachment surface treatment (Corning). 40 x 10^3^ NHDFs were seeded on top of the scaffolds. Cell metabolic activity was measured at days 2, 5, 9 and 14.

### Total protein quantification

Total protein amount was quantified at day 3, 7 and 14 with a Pierce BCA protein assay kit (Thermo Fischer Scientific). Samples of 1x1 cm were cut and placed in ultra-low attachment 24 well plates (Corning). 30 x 10^3^ NHDFs were seeded on each sample in 1 mL of cell culture media. Triplicate of both conditions, scaffolds with and without aligned fibers on the surface, were evaluated. For each condition, blanks were added corresponding to scaffolds without cells to subtract the background signal. Protein extraction was made using mammalian protein extraction reagent (Thermo Fischer Scientific), after rinsed samples 3 times in PBS and freeze them at -80°C. The protocol followed for the quantification was the supplied one by the manufacturer. Briefly, samples were thawed, and the surface scraped. 25 μL of samples were added for triplicate in 96 wells plate. After that, 200 μL of working reagent supplied with the kit were added. A standard curve was prepared at the same time. The wells plate was incubated during 30 minutes at 37°C, following by 5 minutes at room temperature before assessing the absorbance at 562 nm with an Infinite M200 PRO microplate reader (Tecan).

### Cell morphology by confocal fluorescence images

Samples with cells seeded were incubated for 3 and 7 days, at each time point, samples were fixed with 4% Paraformaldehyde (PFA) for 10 minutes. Afterwards, they were rinsed thrice with PBS, and permeabilized with 0.5% Triton X-100 for 5 minutes. Phalloidin (Phalloidin-TRITC) and DAPI (both from Sigma Aldrich) were used for actin and nuclei staining. Images were taken at 10X augments with LSM 800 Zeiss confocal microscope. Actin fiber orientation histograms were obtained with Directionality ImageJ plugin (https://imagej.net/Directionality) from Phalloidin stained images.

### Statistics

Data are presented as mean ± standard deviation of the replicates. Statistical significance was assessed with analysis of variance (ANOVA) with a level of significance of P<0.05. Student’s *t-*test (P < 0.05) was applied at fiber integration into PLC films and suture test when only two conditions were compared.

## Results and discussion

In this study, the fabrication process of patches for RCA is presented. These RCA devices are made of PLC films by electrospraying and PLA nanofibers by electrospinning. The combination of these techniques simplifies the fabrication process just changing parameters like voltage, distance and syringe (polymer solution and concentration) in the same device. Thus, the scale-up of RCA patches is straightforward facilitating laboratory to clinic translation. The wide range of techniques and companies that provide specific electrospinning equipment and solutions for research laboratories and medical device industries is another advantage [26,27].

### Production of organogel PLC films by electrospraying and PLA fibers addition by electrospinning

**Fig 2:**
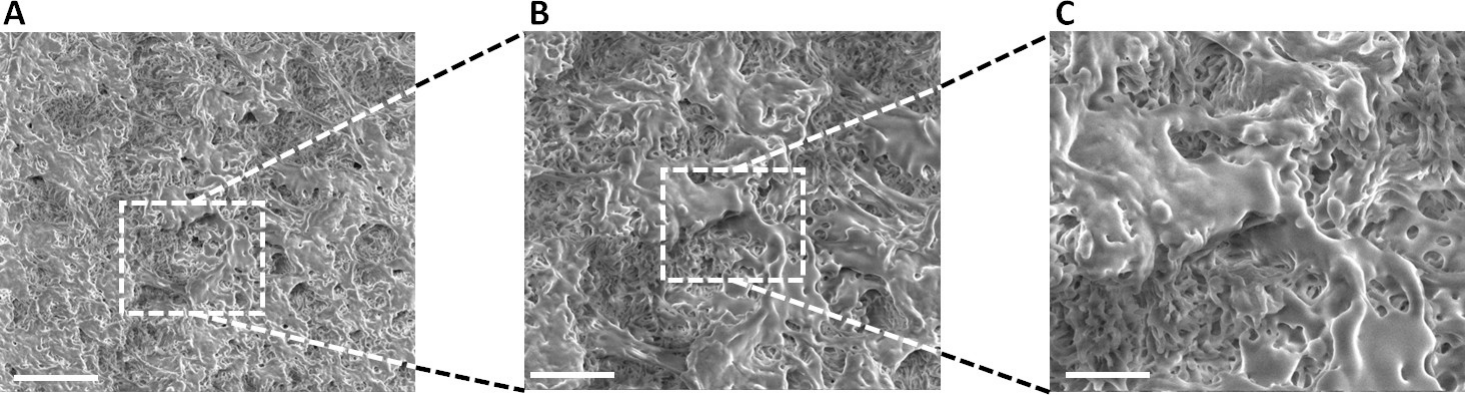
FE-SEM images from PLC films cross section. Inner structure of the PLC organogel film obtained by electrospraying, from A) 5000, B) 6000 and C) 12000 augments. (Scale bars corresponds to 20, 10 and 5 microns for A, B and C, respectively).

The inner microstructure of PLC/EtLac organogels films produced by electrospraying after solvent exchange in water was characterized by FE-SEM [21]. In Fig 2, it is possible to appreciate the fibrous morphology of the film cross section, which validates electrospraying as a new method to obtain a fibrous structure of the PLC films.

**Fig 3:**
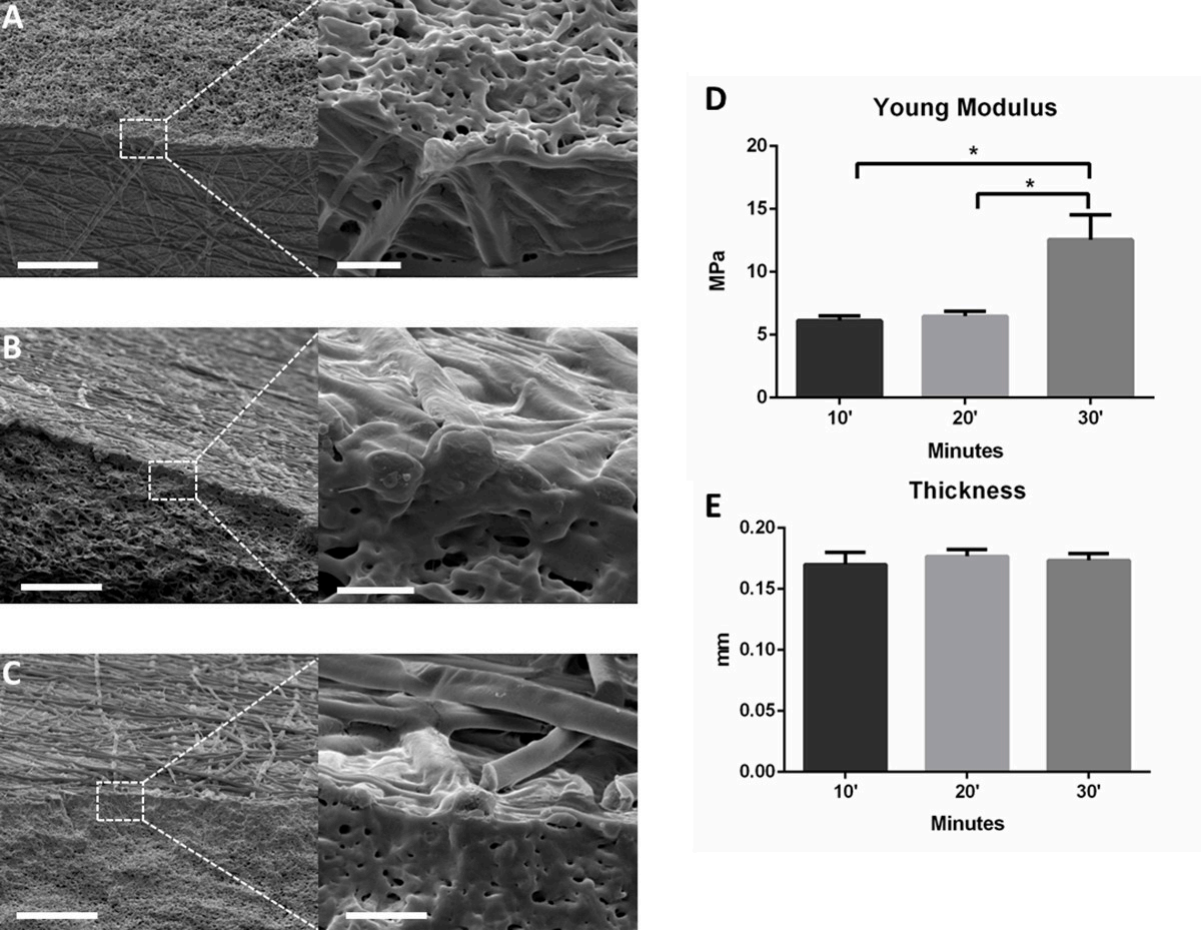
Different production times of electrospun PLA fibers added on a PLC organogel film. FE-SEM cross section images of production PLA fibers on the organogel PLC film during A) 10 minutes, B) 20 minutes and C) 30 minutes (scale bars = 30 microns. Inserts: scale bar = 3 microns). D) Young Modulus and E) thickness measurements of the different conditions (n = 3,*P<0.05).

The addition of PLA nanofibers to the PLC organogel films was a crucial step to reinforce the mechanical properties of the patch, achieving the optimal ones for the RCA application. For this purpose, three different fiber deposition time points were tested (10, 20 and 30 minutes). FE-SEM analysis of the inner structure of the devices shows no differences in terms of fiber adhesion and density (Fig 3A, 3B and 3C). However, a significant increment of the approached Young’s Modulus (14 MPa) was obtained for the 30 minutes condition, in contrast to the 6 MPa obtained for the rest of conditions (Fig 3D and 3E). As expected, the final thickness of the device was not affected by the length of the deposition. Fiber diameter for random PLA fibers was 1.35±0.52μm averaged diameter.

In order to introduce PLA nanofibers between the PLC films and to evaluate the different production times, a sequential alternated production of film/fibers/film was studied. Two different conditions (30 and 60 minutes PLC film production) were tested, whereas the production time of PLA fibers was set constant (20 minutes, as an intermediate time point).

**Fig 4:**
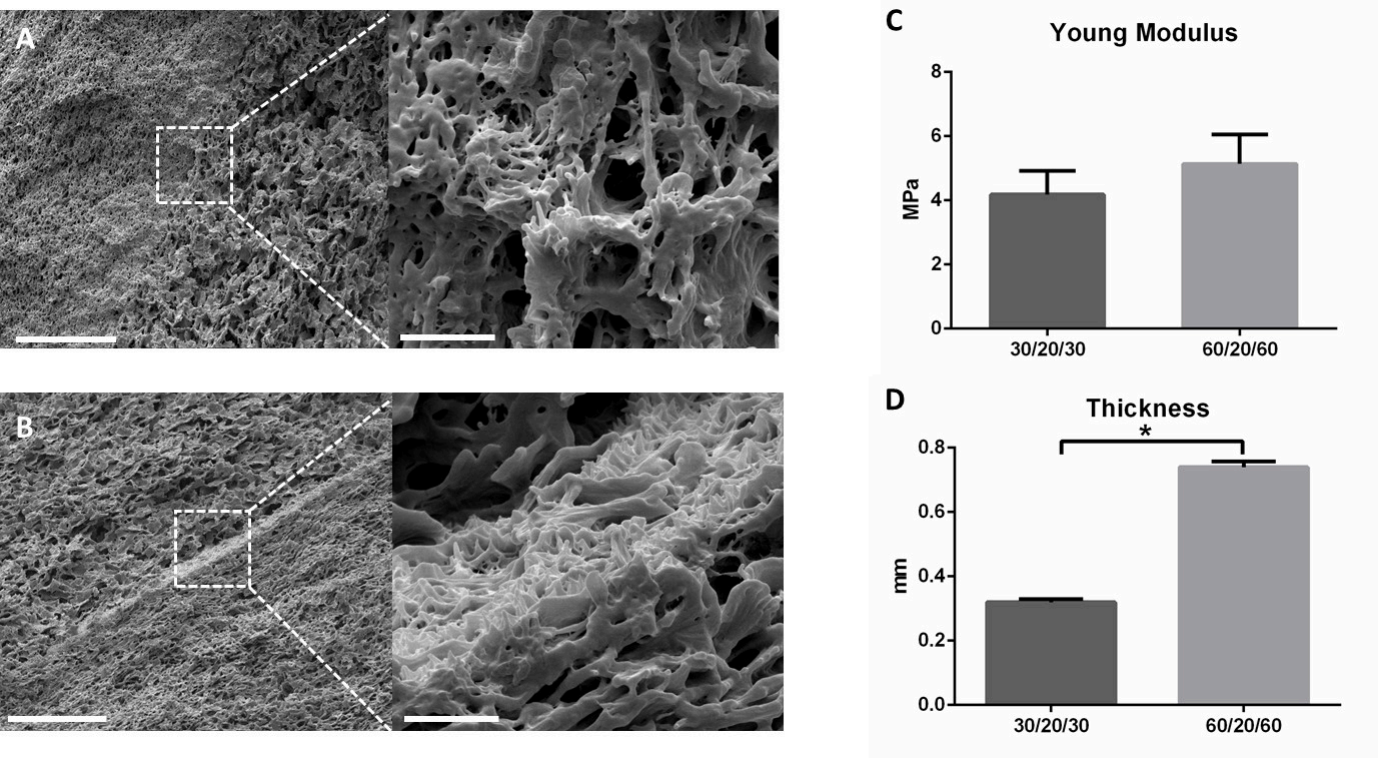
Embedded PLA fibers between two PLC films FE-SEM cross-section images. A) 30 minutes and B) 60 minutes of PLA fibers production (scale bar = 30 microns. Inserts: scale bar = 3 microns). C) Young Modulus and D) thickness measurements of the different conditions (n = 3,*P<0.05).

A complete integration of the fibers for both conditions is observed in (Fig 4A and 4B). However, only is possible to identify union regions of the two films produced, where the PLA fibers are embedded between both PLC films. No significant differences for the approached Young’s Moduli were obtained (around 5 MPa for both conditions) (Fig 4C). In contrast, significant differences were observed increasing PLC film time production: from 0,3 mm for the 30/20/30 condition to 0,65 mm for the 60/20/60 condition (Fig 4D). These results suggest that the PLA fibers layers only affects the mechanical properties of the device, while the PLC film layers has an effect in thickness and the main inner porosity microstructure of the RCA patch.

Thus, the combination different times of production and number of layers of PLC films and PLA fibers allows the fine tuning of the mechanical properties and geometry of the patch.

### Determination of PLC films and PLA fibers layers number

**Fig 5:**
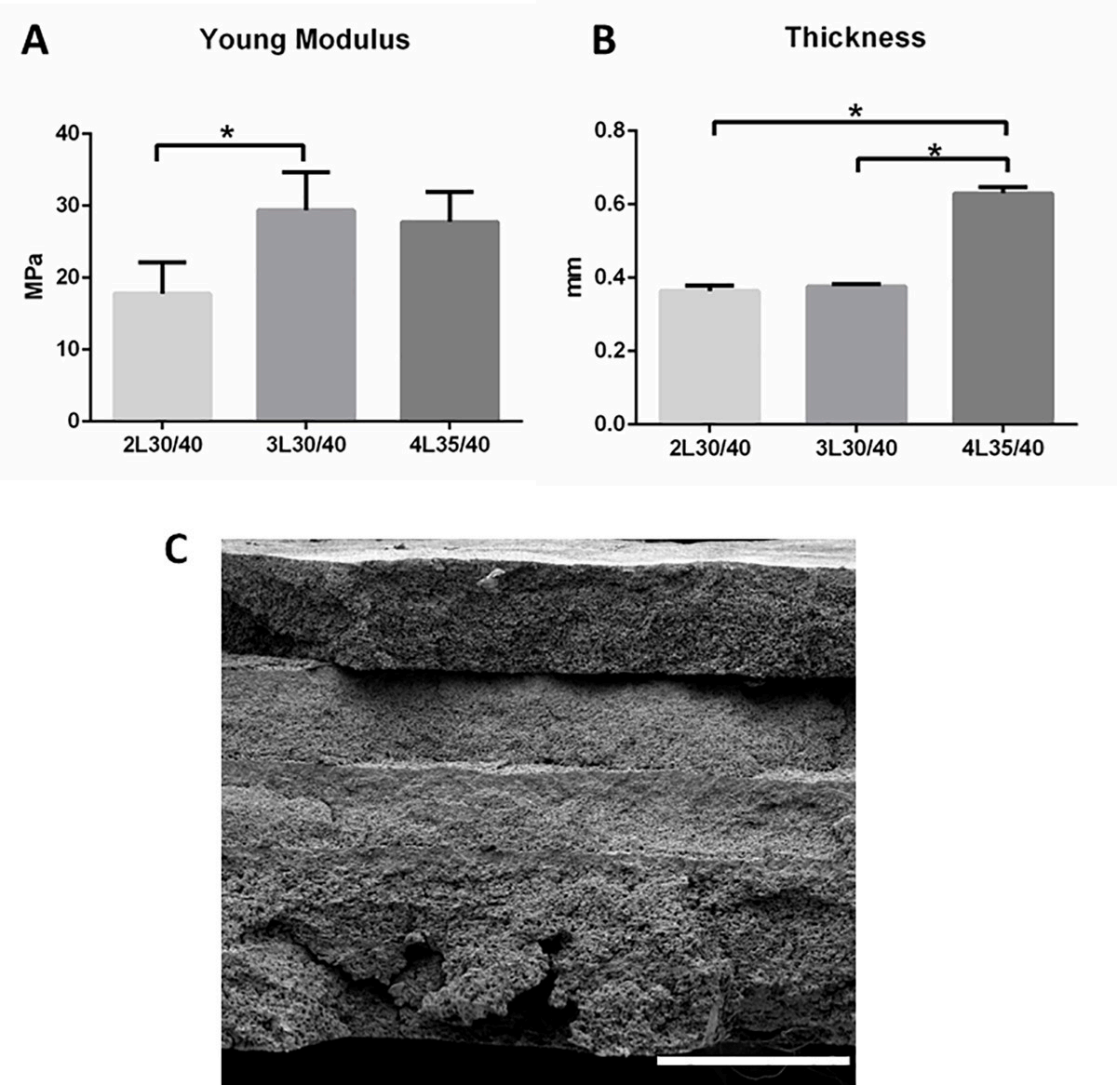
A) Young Modulus and B) thickness measurement for the different conditions (n = 3,*P<0.05). C) FE-SEM images of the inner structure of 4L35/40 (scale bar = 300 microns).

Consequently, different number of PLC films and PLA fibers layers sequentially produced have been studied with the aim to reach similar mechanical properties to other devices reported in the bibliography [28,29]. Three conditions were characterized increasing the number of PLA fibers (see nomenclatures in Table 1). Fig 5A shows the Young Modulus of the different samples. Significant differences were obtained with lower Young’s Modulus at the range of 18MPa for the 2L30/40 condition against 3L30/40 condition (range of 30MPa), no significant differences were obtained between 3L30/40 and 4L35/40. The axial stiffness (calculated from Young Modulus [30]) of the devices were 6.40, 10.54 and 17.34 N/mm for 2L30/40, 3L30/40 and 4L35/40, respectively. Fig 5B. shows the thickness of the three different devices. Although 3L30/40 has similar mechanical properties as 4L35/40, its thickness is around 1.5-fold lower (0.4 and 0.63 mm, respectively). The thickness of the devices can be affected by the number of layers and deposition time. For this reason, PLC films deposition time was increased in 5 minutes and an extra PLA fiber layer was added to maintain the mechanical resistance and increase the thickness (4L35/40 condition). This thickness value of 0.63 mm is similar to RCA commercial patches [31]. SEM images of cross sections were taken for 4L35/40 condition (Fig 5C). The different layers (PLC films and PLA fibers) can be easily identified. Deformation at Yield Strength measured for the three conditions did not show significant differences. As shown in S1A Fig, all conditions are around 2–4% of deformation. S1B Fig show representative elastic region strain-stress curves of the three conditions. This range is compatible with the range of tendon deformation, which at 4% starts the microscopy tearing of tendon fibers [32].

**Table 1:** Nomenclatures relation to the number of layers and time of layer production for each construct studied.

N° PLC film	PLC film Production Time (min/layer)	N° PLA fibers	PLA fibers Production Time (min/layer)	Nomenclature
**3**	**30**	**2**	**40**	**2L30/40**
**4**	**30**	**3**	**40**	**3L30/40**
**5**	**35**	**4**	**40**	**4L35/40**
**5**	**35**	**4**	**40**	F4L35/40*

*Condition with extra 30 minutes of aligned electrospun PLA fibers.

### Suture test

**Fig 6:**
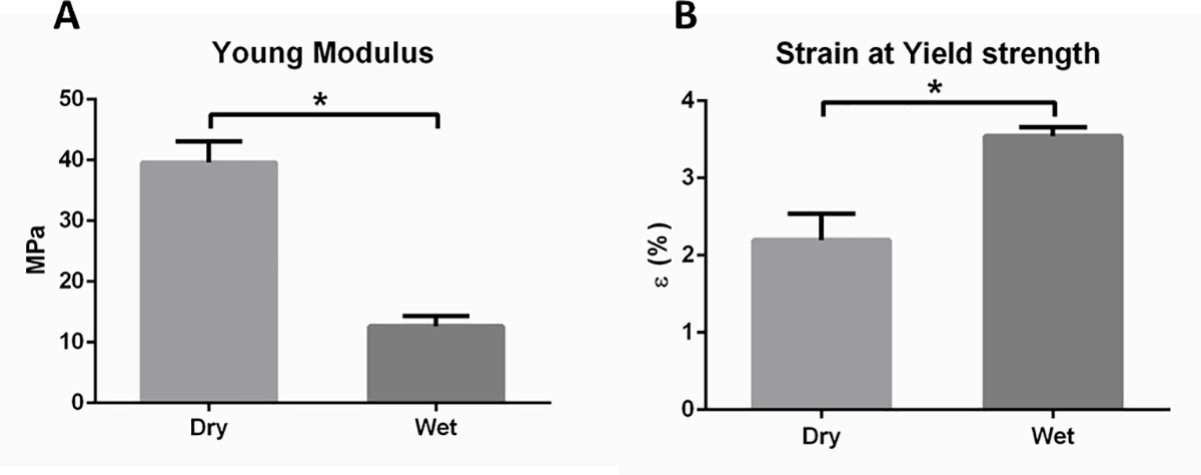
A) Young Modulus and B) Strain at Yield strength obtained for dry and wet conditions of suture assay of 4L35/40 (n = 3,*P<0.05).

A suture test was designed to evaluate the patch behavior [28,33,34]. Two different conditions were evaluated, dry conditions at RT and wet conditions (PBS) at 37°C. As expected, a significant decrease of Young’s Modulus (Fig 6A) and an increase of the strain at the yield strength were obtained for the wet conditions (Fig 6B), due to the higher temperature and liquid media effect. Temperature, in particular for polymers, is an important factor to consider during the material characterization, because it affects directly the mechanical properties and the performance of devices. Despite mechanical properties of patch and suture retention, the tendon-to-bone fixation technique and the patch features plays a crucial role in biomechanics and stability [6,35].

### Biological characterization

**Fig 7:**
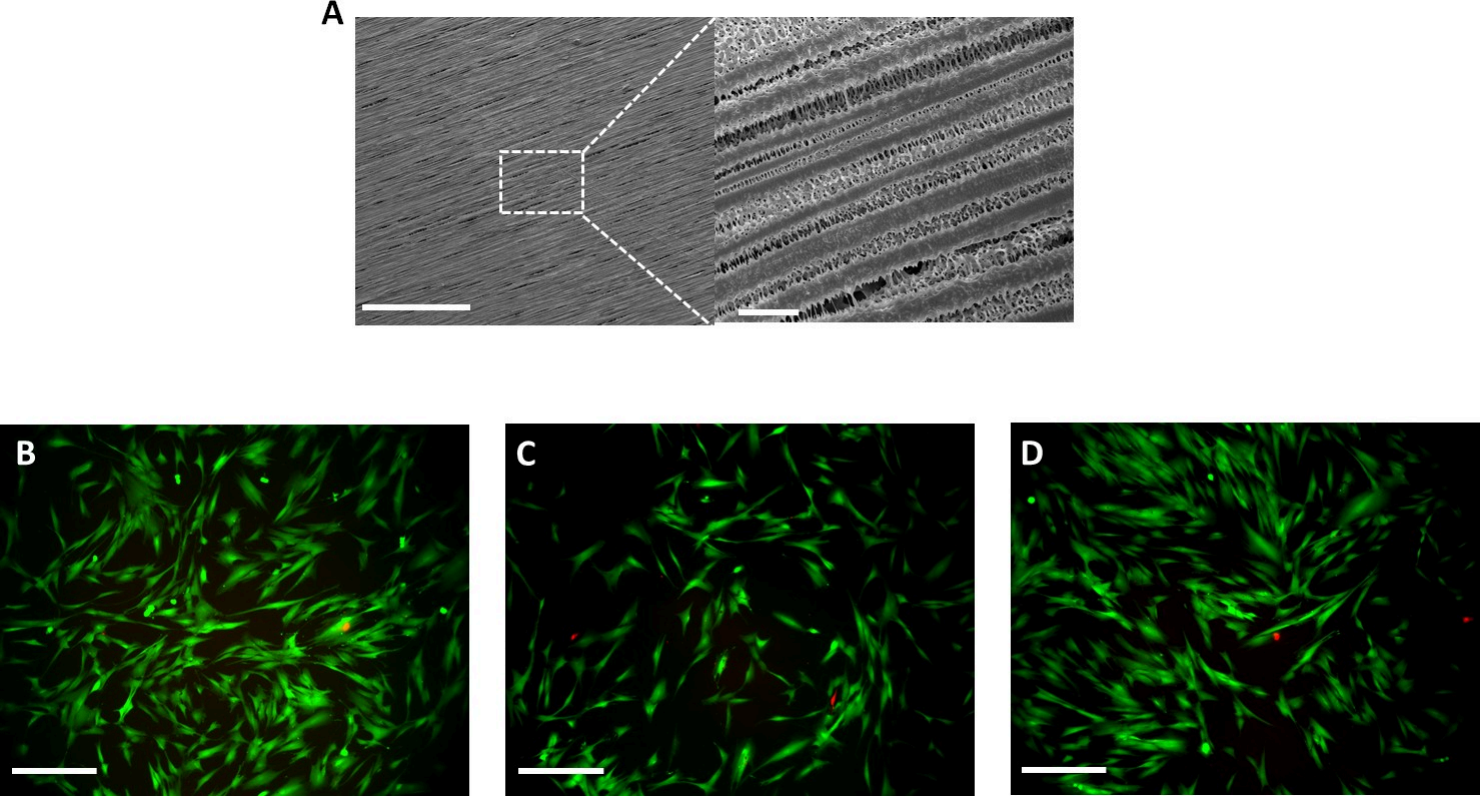
A) FE-SEM images of the surface modification adding electrospun PLA aligned fibers F4L35/40 (scales bar = 300 microns. Insert: scale bar 5 microns). B) Qualitative live/dead assay using the conditioned media from control condition (TCP), C) 4L35/40 and D) F4L35/40 (scale bar = 100 microns).

An ideal RCA patch fabrication method should allow the surface modification to introduce chemical and physical signals on the device to better mimic the tendon structure and bioactivity [36,37]. In this way, electrospinning was also used to produce aligned nanofibers on the surface of the scaffold with 1.47±0.39μm averaged diameter, mimicking the tendon collagen fibers. Tendon has a hierarchical structure organization based on collagen fibers, known as fibrils, aligned to the long axis of the tendon closely packed together [38]. It is known that aligned patterns affect cells phenotype [39].Yin et al. found that aligned electrospun fibers promote tendon stem/progenitor cells differentiation to tendon linages [40]. This topography effect has been confirmed also with *in vivo* assays, where random fibers induced Mesenchymal Stem Cells (MSCs) to bone linage and aligned fibers to tendon linage [41]. Fee et al. showed that genes related to actin production, polymerization and focal adhesions were upregulated when fibroblast were seeded on aligned electrospun fibers [42]. Due to that, one condition with aligned PLA fibers on the surface (noted as F4L35/40 at Table 1) was added to the previous condition selected (4L35/40) for the biological characterization. Mechanical properties obtained for F4L35/40 condition were no significatively different from 4L35/40 (28.5MPa approached Young Modulus). FE-SEM images of the surface for the fibers condition were taken (Fig 7A). The effect of the surface pattern to cell metabolic activity and protein production was studied comparing the condition of 4L35/40 and F4L35/40 for all the biological assays.

First step was to check whether scaffolds had any toxicity effect to NHDFs cultures, for example, as a result of solvent residues after precipitation process. In Fig 7B, 7C and 7D, cell viability shows above 90% of cells are alive (green cells alive, red cells dead). Thus, for the LDH cytotoxic assay prolonged in time, until 9 days (S2 Fig), lack of toxicity was observed for both conditions.

**Fig 8:**
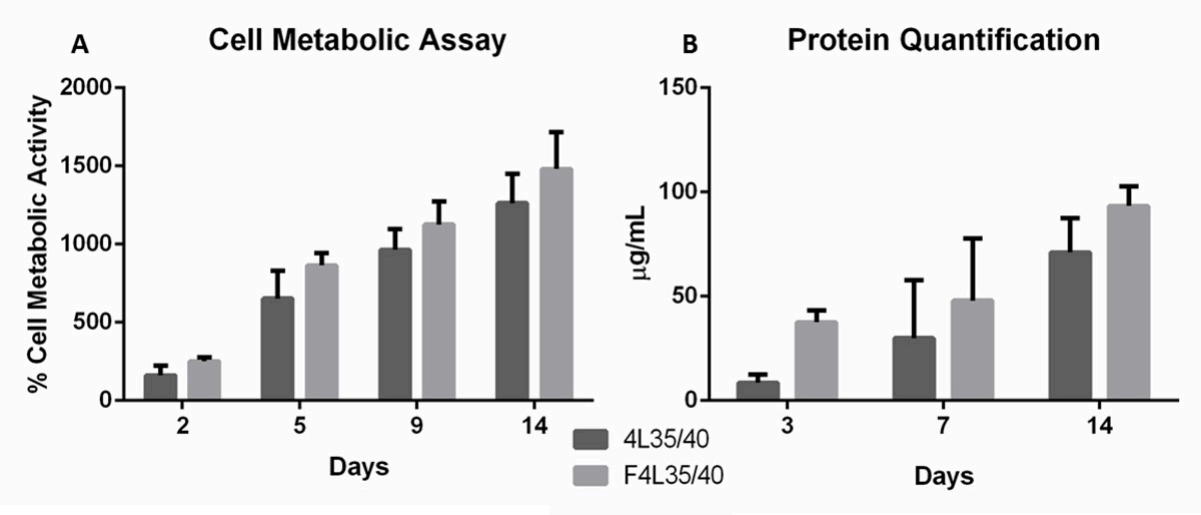
A) Cell metabolic activity, data is referenced in percentage respect to the values obtained at 3 hours (n = 6,*P<0.05). B) Total protein quantification (n = 3,*P<0.05).

Cell metabolic assay on the scaffold surface was evaluated seeding NHDFs on 4L35/40 and F4L35/40. Results are shown as a percentage of the measure done after 3 hours of seeding. No significant differences were obtained between both conditions for all the measured time points for the cell metabolic assay (Fig 8A). Both conditions have a positive linear slope during the assay. It has already been shown that there is no correlation between fibers direction and fibroblasts proliferation [43].

The amount of proteins produced by cells was also quantified for both conditions (Fig 8B). No significant differences were obtained in protein production between F4L35/40 and 4L35/40 conditions from days 3 to 14. Even though, the benefits for tendon/ligament applications of fiber alignment on ECM proteins production have been previously reported. C. Hunt et al. reported more collagen production by fibroblasts seeded on aligned fibers than seeded on random orientation fibers [44]. Also Thomas K. et al. found an increase of production of Collagen I at day 7 and Collagen III at day 14 by MSCs seeded on aligned fibers than random [45]. In addition, Wang et al. showed that aligned culture produce also an aligned collagen matrix, like tendon structure [46]. More assays are needed to study the biological effect of adding aligned fibers on the RCA device, and their benefits. The possibility of modulating the production of ECM proteins like collagen type III and I, which are characteristics of the second and third tendon healing stages [47,48], by the surface pattern might be a way to enhance the tendon healing process.

**Fig 9:**
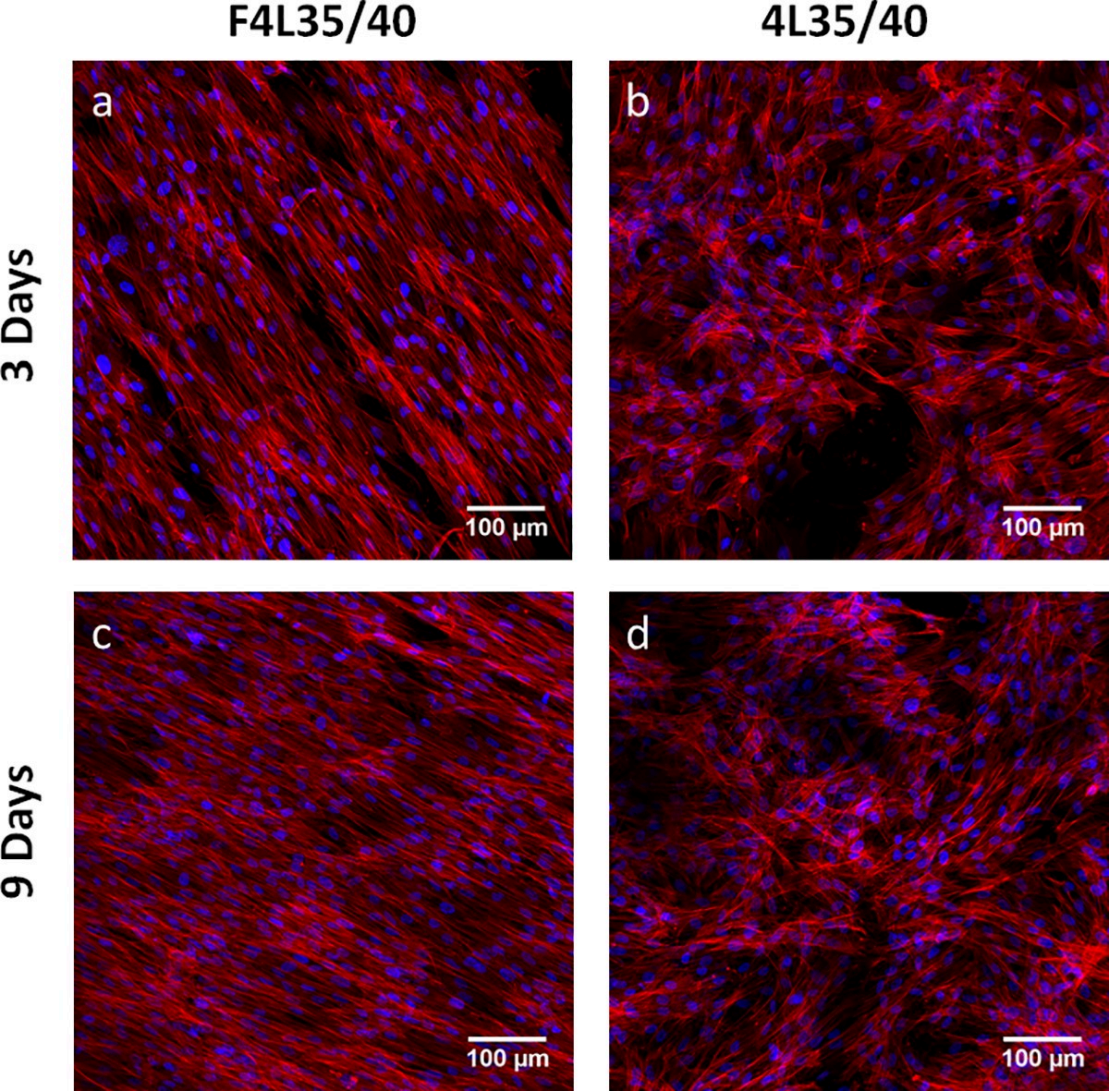
Confocal images of NHDFs A) after 3 days cultured on F4L35/40, B) after 3 days cultured on 4L35/40, C) after 7 days cultured on F4L35/40 and D) after 7 days cultured on 4L35/40. Actin stained with Phalloidin-TRITC (red) and nuclei stained with DAPI (blue).

Finally, the effect of the aligned fibers condition in morphogenesis was also studied by fluorescent confocal images. It is known that aligned patterns also affect the cells’ morphology following fibers orientation [49]. At Fig 9, it is possible to observe the effect of surface pattern to cell morphology after 3 and 7 days of culture. After 3 days of culture, it is possible to appreciate the aligned morphology of NHDFs seeded on F4L35/40 (Fig 9A) in contrast with rounded shape morphology for NHDFs seeded on 4L35/40. After 7 days, both conditions are completely colonized by NHDFs, maintaining their morphology observed at day 3 (Fig 9C and 9D). Histograms of actin fibers orientation were obtained from Phalloidin staining from confocal images of NHDFs (S3A and S3B Fig). As it is shown, the aligned fiber pattern had a contact-guide effect on the cells after 7 days cultured on F4L35/40 (S3C Fig), with a Full width at half maximum of approximately 22° (gaussian fitted curve). No actin orientation was detected for NHDFs cultured on 4L35/40 (S3D Fig). Therefore, these results supported with the bibliography findings, suggest that aligned fibers patterns affect their morphology and could influence the ECM topography mimicking the tendon tissue structure, and could be a promising strategy to improve surface bioactivity for a rotator cuff augmentation device.

## Conclusions

Here we presented a novel method to produce organogel PLC/EtLac films by electrospraying, and also characterized their mechanical properties improvement by the addition of PLA fibers using electrospinning. Combining the two methods made possible the fabrication of a degradable synthetic composite patch for RCA, obtaining the optimal configuration, 4L35/40 condition, for a rotator cuff augmentation device application. Moreover, surface patch pattern has been modified by adding electrospun aligned PLA fibers mimicking tendon structure to promote the successful biological response, achieving a cell alignment morphology like tendon tissue. *in vivo* experiments are needed to confirm the adequate performance of this new device for tendon applications. However, the scale-up for industrial production looks promising due to the simplicity and versatility of the set-up here proposed.

## Supporting information

S1 Fig A) Deformation at Yield Strength (n = 3, *P<0.05). B) Representative Strain-Stress curves adjusted to highlight elastic regions.
(TIF)

S2 Fig Results obtained for the cytotoxicity assay with conditioned media. Values are normalized by the control condition (cells with media that were not in contact with materials). (n = 4, *P<0.05).
(TIF)

S3 Fig Actin staining of NHDFs after 7 days cultured on F4L35/40 A), and on 4L35/40 B). Fiber orientation histogram of NHDFs actin staining after 7 days cultured on F4L35/40 C), and on 4L35/40 D).
(TIF)

S1 File RCA device fabrication protocol. (DOCX)
