# Supplementary figures and images for: Development of a novel automatable fabrication method based on electrospinning co electrospraying for rotator cuff augmentation patches
Source: PLoS One. 2019 Nov 14;14(11):e0224661. doi: 10.1371/journal.pone.0224661 (PMC6855444; doi:10.1371/journal.pone.0224661)

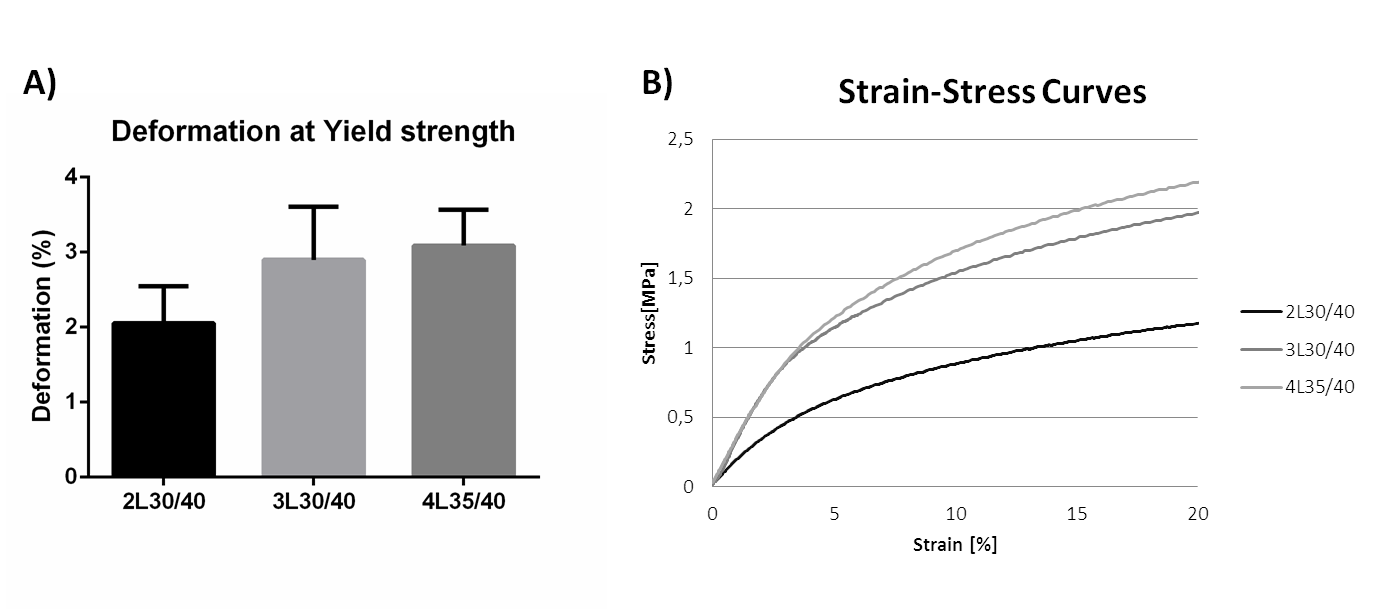

Supplement: S1 Fig — A) Deformation at Yield Strength (n = 3, *P<0.05). B) Representative Strain-Stress curves adjusted to highlight elastic regions. (TIF) [file pone.0224661.s001.tif]

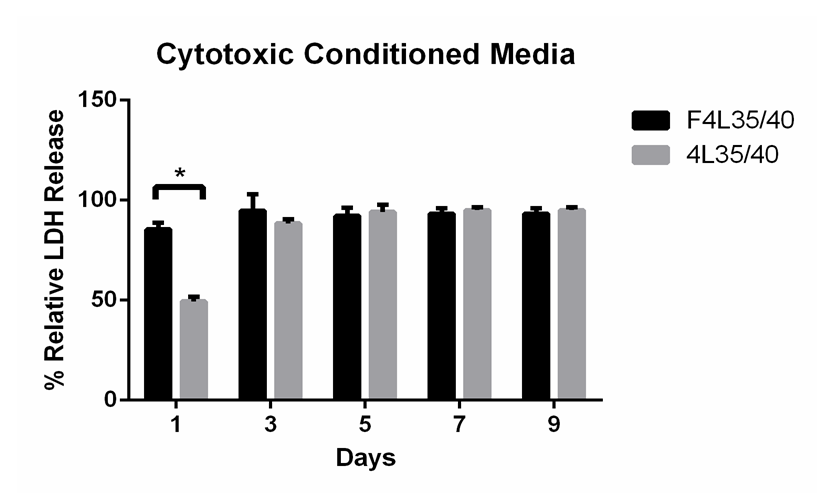

Supplement: S2 Fig — Values are normalized by the control condition (cells with media that were not in contact with materials). (n = 4, *P<0.05). (TIF) [file pone.0224661.s002.tif]

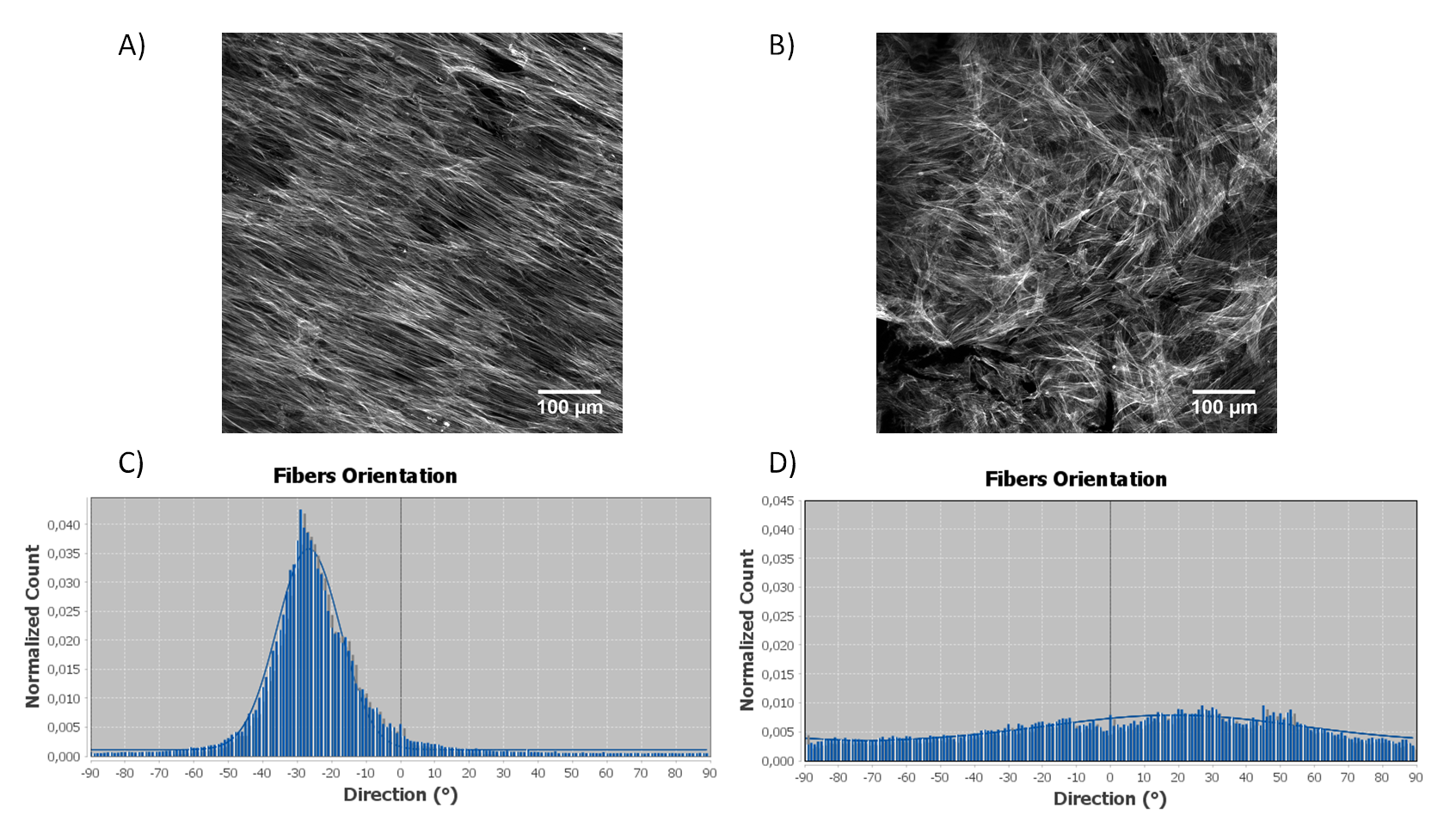

Supplement: S3 Fig — Actin staining of NHDFs after 7 days cultured on F4L35/40 A), and on 4L35/40 B). Fiber orientation histogram of NHDFs actin staining after 7 days cultured on F4L35/40 C), and on 4L35/40 D). (TIF) [file pone.0224661.s003.tif]
