# Supplementary material for: Development of a novel automatable fabrication method based on electrospinning co electrospraying for rotator cuff augmentation patches
Source: PLoS One. 2019 Nov 14;14(11):e0224661. doi: 10.1371/journal.pone.0224661 (PMC6855444; doi:10.1371/journal.pone.0224661)
Supplement: S1 File — (DOCX) [file pone.0224661.s004.docx]

**Polymer solutions**

PLC solution preparation:

- Weight the amount of PLC and take the volume of Ethyl Lactate to obtain a 15% w/v. For example, to prepare 50 mL of PLC solution, 7.5g of PLC were weighted and placed into 50 mL of Ethyl Lactate.
- Mix them into a glass bottle. Set the bottle into a temperature water bath (70ºC) and under magnetic stirring.
- Leave them overnight.
- At the moment of use it, it is necessary to heat the solution at 70ºC.

PLA solution preparation:

- Weight the amount of PLA and take the volume of Trifluoroethanol to obtain a 4% w/w. For example, to prepare 12 mL of PLA solution, 0.66g of PLA were weighted and placed into 12 mL of Trifluoroethanol.
- Mix them into a glass vial. Set the vial under orbital agitation.
- Leave them in agitation overnight at room temperature (RT).

**Fabrication Process**

The fabrication process of the construct is the result of producing PLC films and PLA fibers one after the other. Always the first and the last layers are PLC films. For the surface modification to obtain an aligned pattern, aligned PLA nanofibers can be done as a first layer. After finishing the construct, it is placed into a water bath for Ethyl Lactate solvent exchanging (FIGURE 1). EVA gum can be used to delimitate the area where the construct will be done. For that, it is necessary to put EVA gum layers on the aluminum foil when the collector is prepared before to start. Then, EVA gum is leaved on the collector during the entire fabrication process (FIGURE 2).

Electrospraying PLC film production:

- The amount of PLC solution needed is placed into a syringe. It is better not to use a syringe bigger than 10mL because the solution must be hot, and for bigger volumes, the solution cools quickly.
- The syringe is placed into the pump and metallic tip (0.25mm inner diameter) is connected to the power supply.
- The rotor collector is covered with aluminum foil and connected to grown. EVA gum can be used to delimitate the film production in an area of the collector.
- Set the parameters for the electrospraying process: voltage 8KV, pump rate: 10mL/h, rotator speed 90rpm, and distance tip to collector: 10-18cm.
- Distance tip to collector can be adjusted from 10 to 18 cm depending the size of the electrospraying cone formed.

Electrospinning PLA random fibers production:

- The amount of PLA solution needed is placed into a syringe at RT.
- The syringe is placed into the pump and metallic tip (0.25mm inner diameter) is connected to the power supply.
- The rotor collector is covered with aluminum foil and connected to grown. EVA gum can be used to delimitate the film production in an area of the collector.
- Parameters for the electrospraying process are the following: voltage 8KV, pump rate: 0.5mL/h, rotator speed 90rpm and distance tip to collector: 12cm.

PLA aligned fibers production (surface):

- In case of surface modification to obtain an aligned nanofibers pattern, this layer must be done as the first one. Due to the high rotation speed needed to produce it, layers done previously will be destroyed.
- The amount of PLA solution needed is placed into a syringe at RT.
- The syringe is placed into the pump and metallic tip (0.25mm inner diameter) is connected to the power supply.
- The rotor collector is covered with aluminum foil and connected to grown. EVA gum can be used to delimitate the film production in an area of the collector.
- Parameters for the electrospraying process are the following: voltage 8KV, pump rate: 0.5mL/h, rotator speed 1200rpm and distance tip to collector: 12cm.

**Mechanical Characterization**

For mechanical testing, geometry of specimens is 3x1cm. Three types of mechanical characterization are detailed: tensile test, suture test in dry conditions (FIGURE 3a) and suture test in wet conditions (FIGURE 3b). Equipment used is Zwick/Roell BT1 FR0.5TN.D14.

Tensile test:

- Set the parameters for the testing machine: speed: 10mm/min, preload: 0.1N and preload speed: 5mm/min.
- Sandpaper is added to the clamps to fix better the specimens.

Suture test in dry conditions:

- Set the parameters for the testing machine: speed: 10mm/min, preload: 0.1N and preload speed: 5mm/min.
- The specimen is fixed to one clamp for the lower site and to the upper by a suture wire as it is shown in FIGURE 3c. Suture wire used is made of Polyglycolic acid, USP 2, metric 5, of Aragó brand.
- Sandpaper is added to the lower clamps to fix better the specimens.

Suture test in wet conditions:

- Set the parameters for the testing machine: speed: 10mm/min, preload: 0.1N and preload speed: 5mm/min.
- Specimen is fixed to one clamp for the lower site and to the upper by a suture wire. Suture wire used is made of Polyglycolic acid, USP 2, metric 5, of Aragó brand.
- Sandpaper is added to the lower clamps to fix better the specimens.
- In that case, specimen, clamp and suture is set into a phosphate-buffered saline (PBS) bath provided by Zwick-Roell. To heat the bath, a coil of silicone tube is placed inside the bath connected to a recirculate water heater (E100, Lauda).

**Figures**


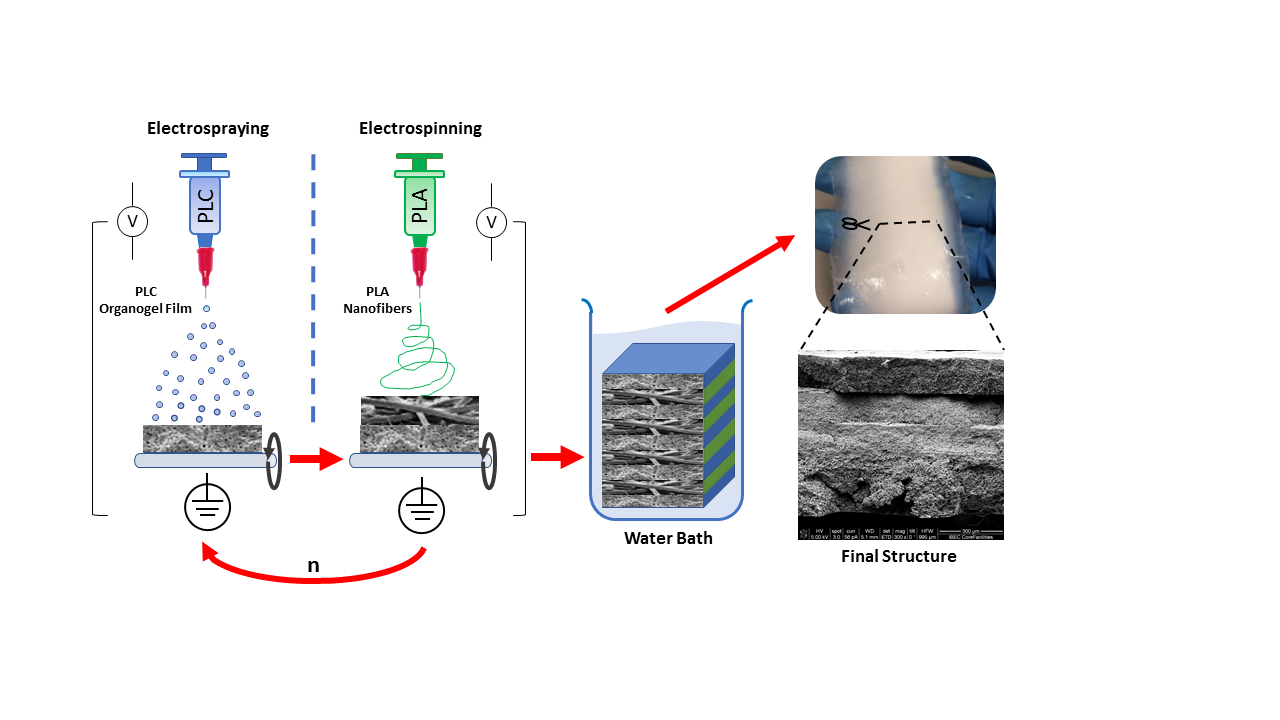


Figure 1. Schematic workflow procedure combining electrospraying (forming PLC films) and electrospinning (forming PLA fibers).


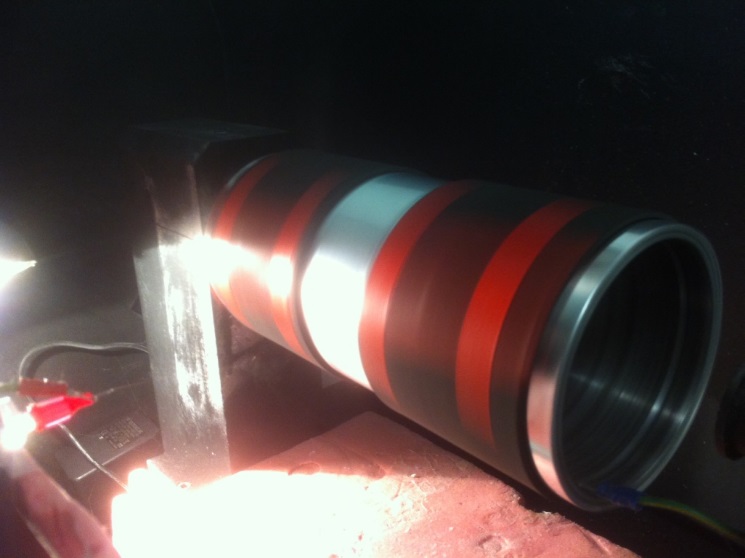


Figure 2. Example of using EVA gum (black) to delimitate the collector surface during the fabrication process.


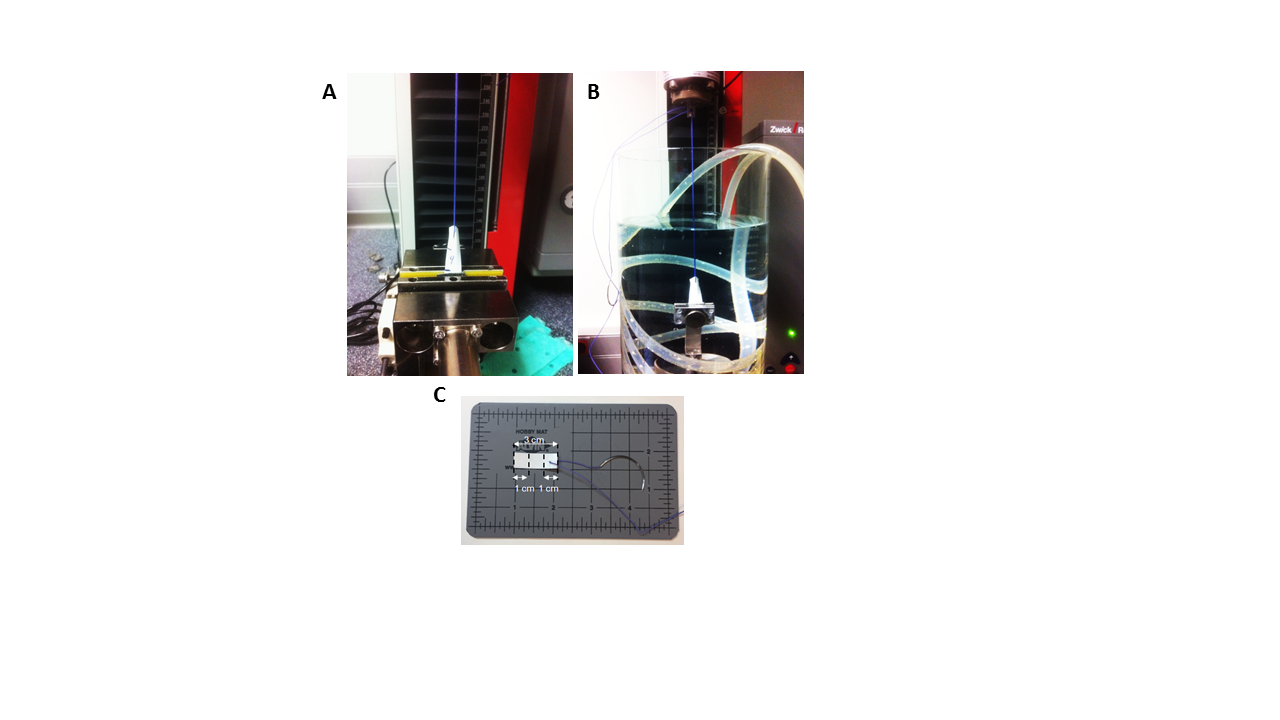


Figure 3. Suture tensile test assay. Image from suture test assay settings used to perform the test in A) dry conditions and B) wet conditions. C) Geometries used to fix the loop suture to the specimens.
